# Cyber security Enhancements with reinforcement learning: A zero-day vulnerabilityu identification perspective

**DOI:** 10.1371/journal.pone.0324595

**Published:** 2025-05-27

**Authors:** Muhammad Rehan Naeem, Rashid Amin, Muhammad Farhan, Faisal S. Alsubaei, Eesa Alsolami, Muhammad D. Zakaria

**Affiliations:** 1 Department of Computer Science, University of Engineering and Technology Taxila, Taxila, Pakistan; 2 Faculty of Informatics and Computing, University Sultan Zainal Abidin, Besut Campus, Besut, Terengganu, Malaysia; 3 Department of Computer Science, COMSATS University Islamabad, Sahiwal Campus, Pakistan; 4 Department of Cybersecurity, College of Computer Science and Engineering, University of Jeddah, Jeddah, Saudi Arabia; Cardiff Metropolitan University - Llandaff Campus: Cardiff Metropolitan University, UNITED KINGDOM OF GREAT BRITAIN AND NORTHERN IRELAND

## Abstract

A zero-day vulnerability is a critical security weakness of software or hardware that has not yet been found and, for that reason, neither the vendor nor the users are informed about it. These vulnerabilities may be taken advantage of by malicious people to execute cyber-attacks leading to severe effects on organizations and individuals. Given that nobody knows and is aware of these weaknesses, it becomes challenging to detect and prevent them. For the real-time zero-day vulnerabilities detection, we bring out a novel reinforcement learning (RL) methodology with the help of Deep Q-Networks (DQN). It works by learning the vulnerabilities without any prior knowledge of vulnerabilities, and it is evaluated using rigorous statistical metrics. Traditional methods are surpassed by this one that is able to adjust to changing threats and cope with intricate state spaces while providing scalability to cybersecurity personnel. In this paper, we introduce a new methodology that uses reinforcement learning for zero-day vulnerability detection. Zero-day vulnerabilities are security weaknesses that have never been exposed or published and are considered highly dangerous for systems and networks. Our method exploits reinforcement learning, a sub-type of machine learning which trains agents to make decisions and take actions to maximize an approximation of some underlying cumulative reward signal and discover patterns and features within data related to zero-day discovery. Training of the agent could allow for real-time detection and classification of zero-day vulnerabilities. Our approach will have the potential as a powerful tool of detection and defense against zero-day vulnerabilities and probably brings significant benefits to security experts and researchers in the field of cyber-security. The new method of discovering vulnerabilities that this approach provides has many comparative advantages over the previous approaches. It is applicable to systems with complex behaviour, such as the ones presented throughout this thesis, and can respond to new security threats in real time. Moreover, it does not require any knowledge about vulnerability itself. Because of that, it will discover hidden weak points. In the present paper, we analyzed the statistical evaluation of forecasted values for several parameters in a reinforcement learning environment. We have taken 1000 episodes for training the model and a further 1000 episodes for forecasting using the trained model. We used statistical measures in the evaluation, which showed that the Alpha value was at 0.10, thereby indicating good accuracy in the forecast. Beta was at 0.00, meaning no bias within the forecast. Gamma was also at 0.00, resulting in a very high level of precision within the forecast. MASE was 3.91 and SMAPE was 1.59, meaning that a very minimal percentage error existed within the forecast. The MAE value was at 6.34, while the RMSE was 10.22, meaning a relatively low average difference within actuals and the forecasted values. Results The results demonstrate the effectiveness of reinforcement learning models in solving complex problems and suggest that the model improves in accuracy with more training data added.

## Introduction

The problem with zero-day bugs is huge in the world of cyber security and requires immediate solutions as the attacker will take such bugs and cause real harm to organizations, individuals, and sprawling communities. This research work therefore resulted in the development of the method of immediately discovering zero-day vulnerabilities using reinforcement learning. It roughly defines the subdomain of machine learning where some particular action is being done by machines to act and make some decisions in a particular environment so that the total reward signal generated should be maximum. The DQN agent learns through the reward signal in terms of reinforcement regarding how it needs to decide concerning zero-day vulnerability detection. The reward signal is determined by the correctness of predictions made by the agent to ‘correct’ the agent along the right path to make more correct predictions. For this paper, the reward signal was calculated proportional to the number of correctly detected zero-day vulnerabilities in the time they were found. This is useful for numerous controls, optimization, and decision-making problems in complex dynamic environments. Reinforcement learning is an application area of cyber security. For example, systems may be expected to detect and counter malware. Malware is short for malicious software and is software that is designed to get into your computer’s system without your knowledge or consent. Multiple variants exist for malware, including viruses, worms, trojans and ransomware. Most cases of malware are, of course, malicious, used to steal confidential information, disrupt operations, or access systems without permission. The consequences of a successful attack are too grave to be ignored and must be won by security professionals and researchers.

A zero-day vulnerability happens when there’s a flaw in software that no one’s noticed or fixed yet, making it a sweet spot for hackers to attack. These vulnerabilities are dangerous because they get used by bad actors before a fix is available. In this paper, we’re talking about a new method to find and fight malware using reinforcement learning. It’s about training a program, called an agent, to recognize patterns that show something might be malicious. We give this agent a bunch of data, both safe and harmful. Over time, the agent starts to see which features are usually linked to malware. Once it’s trained, the agent can check out new data it hasn’t seen before and figure out if it’s risky or not. The agent learns by making predictions and getting feedback, and it keeps improving as it goes along, getting better at telling what’s dangerous.

The work’s key contribution is in developing a DQN based RL approach for detecting zero-day vulnerabilities. In contrast, unlike traditional methods, the DQN agent is able to learn from experience; another similar technique was not needed to balance exploration and exploitation; and the agent is able to handle large, complex datasets. The patterns that are suggestive of vulnerabilities are identified, and the accuracy increases with time, and it becomes a robust and adaptive solution for real time threat detection.

Over the years, several techniques have been applied to identify zero-day vulner-abilities: In particular it involves signature-based detection, anomaly-based detection as well as machine learning approaches. Signature based detection is only dependent on the knowledge of previous threats and their associated exploits. For example, this approach doesn’t have the ability to detect the new kind of threat or attack, so it’s not capable of detecting well-known and accurate information intending to protect it and does not see the zero-day if has no signature of a threat. One of the anomaly detection approaches attempts to detect the activity that is taking place in a system in an abnormal way, which could mean that a zero-day vulnerability is happening.

The main advantage is to discover previously unknown threats, but the model of normal system behavior must be accurate in practice [1]. Therefore, machine learning-based methods have already bound signature-based and anomaly detection methods. Specifically, such methods rely on algorithms using the step of classifiers such as decision tree classification, random forests and neural networks for the creation of unique respective patterns in the large datasets of vulnerabilities and exploits that may or may not indicate the existence of zero-day vulnerabilities.

Probably the most basic mathematical concept in RL, the Bellman equation, specifies how to express the value of taking a particular action in each state-that is defined as the expected sum of discounted rewards that would be obtained by taking a particular action in that state. The Bellman equation can be described as a recursive relationship of value between that of the current state and all the future states, whereby values of one state could be estimated by the values of other states. During the detection of zero-day vulnerability using the DQN agent, the Bellman equation would thus be used in approximating how much reward a player would get from taking a specific action in a certain state in the future. In this case, the state would represent the state of the system at any given time and the action would represent that of which vulnerability to look at. The update of the action-value function approximating the value of taking a given action in any state is done using expected future rewards.

The backpropagation algorithm is the supervised training procedure used in the training of a neural net. Similar to what was done in this experiment, the application of backpropagation is performed on the neural net describing the action-value function introduced for the DQN agent. This algorithm’s application utilizes an implementation of gradient descent toward minimizing the differences between the estimated action-value function and the target action-value function, which has been updated according to the Bellman equation. Hyperparameters are parameters in a machine learning model that are set before training and determine the behaviour of the model. In the implementation of the DQN agent for zero-day vulnerability detection, hyperparameters such as the learning rate, the discount factor, and the exploration rate are set before training and determine the speed and accuracy of the training process. The selection of appropriate hyperparameters is an important aspect of the implementation and can have a significant impact on the performance of the DQN agent.

An approach in malware identification based on reinforcement learning might, therefore, turn out to be one of the powerful and efficient methods through which malware detection and its fighting may be made to happen. In this case, successful training of the reinforcement learning agent can identify characteristic traits of malware, and a built system adaptable to changing conditions makes accurate predictions about the presence of malware in data [2]. It may offer critical benefits to security specialists and researchers in this discipline. To fulfil the objective of the current study, this method seems to train a DQN agent on an archive of vulnerabilities and exploits that need surveillance pre-cursor patterns of zero day vulnerabilities. This is used to test a new set of vulnerabilities in order to verify the ability of the agent for reliability in detecting zero-day vulnerabilities with high accuracy in real-world cases. These are the outcomes of the research work, showing very important potential in highlighting the usefulness of using reinforcement learning techniques for problem-solving methodologies in cybersecurity and the enhancement of security levels within digital systems [3].

Then, the strengths of using a DQN agent in the context of zero-day vulnerability detection would therefore include learning experience, so that it becomes progressively better and more accurate with time, appropriate to large and even complex states, and proper balance between exploration and exploitation of maximal rewards received. The trained DQN agent identifies the data that it encounters and learns patterns of data within the system with respect to zero-day vulnerabilities such that it may form more accurate predictions over time. Moreover, using neural networks in the DQN agent, it endures vast and complex state spaces that are difficult to model with traditional approaches [4].

Other approaches that could be applied in the case of zero-day vulnerability detection include supervised learning techniques such as decision trees or random forests, but also unsupervised learning techniques like clustering or anomaly detection. Such approaches perform very badly for large and complex state spaces and thus would not be able to learn from experience nearly as well as a DQN agent generally. Despite these limitations, in this work, a DQN agent has been selected for zero-day vulnerability detection because this agent can handle large and highly complex state spaces, learn from experience and balance exploration and exploitation simultaneously. DQN agents hold promises with respect to more accurate and efficient zero-day vulnerability detection than the existing alternatives and are, therefore, an exciting approach to tackle this important cyber security problem [5,6].

Zero-day vulnerabilities are the most dangerous issues in cybersecurity -the unknown security weaknesses that an attacker uses to achieve unauthorized access to systems or disrupt operations. Thus, zero-day vulnerabilities must be found and debugged by security experts and researchers because the impact of an attack can be devastating, if successful.

As such, we are trying to create a quick and efficient way in order to spot and identify zero-day vulnerabilities in real time. At the heart of our interest is building a system that can automatically adapt to new conditions and detect unknown vulnerabilities emerging to protect systems and networks against potential attacks. Without wasting our time further, let’s denote the task of Z or zero finding vulnerabilities as Z. Denote by D the set of malware and benign samples, and f(D) a function that maps the input dataset to a (zero) day vulnerability prediction. Our goal is to learn the function f in such a way that f(D) was the most accurate fit. A reinforcement learning agent (in this case represented through DQN algorithm), interacts with the environment taking action and getting reward according to the correctness of its predictions. The reward signal R is simply the measure if the prediction accuracy of the function f(D). The task of the DQN is to learn a mapping from the input dataset to the optimal action that maximizes the reward signal R, thus solving the problem of detecting zero-day vulnerabilities Z. Mathematically, the problem can be formulated as shown in Eq (1):


$$\max_{f}R(f(D))$$ (1)

s.t.f is learned by the DQN agent.

The research addresses the following questions: how effective is a reinforcement learning-based approach at identifying zero-day vulnerabilities compared to traditional methods? Can a reinforcement learning-based approach adapt to changing conditions and identify new, previously unseen vulnerabilities? What are the most important features for a reinforcement learning-based approach to consider when classifying data as malicious or benign?

The proposed approach in this study, using a DQN agent for zero-day vulnerability detection, builds upon the strengths of previous methods by combining the ability to detect previously unknown threats with the ability to learn from experience. The DQN agent is trained on a dataset of vulnerabilities and exploits to identify patterns that are indicative of zero-day vulnerabilities, and it can continually improve its accuracy over time as it is exposed to new data. This approach offers the potential for more accurate and efficient zero-day vulnerability detection compared to previous methods.

The organization of this research paper is divided into six main sections: Introduction, Literature Review, Proposed Methodological Framework, Results and Discussion, Conclusion & Future Work, and References. The Introduction provides a background of the research topic and sets the context for the study. The Literature Review section critically evaluates the existing knowledge on the topic and identifies the research gap that the current study aims to address. The Proposed Methodological Framework section describes the research design and methods used to carry out the study. The Results and Discussion section presents the findings of the study and interprets their significance. The Conclusion summarizes the main findings and highlights their implications, while the Future Work section outlines potential areas for further research. Cited references are listed at the end of this research paper.

## Literature review

In this section, various papers have discussed and presented novel approaches for identifying zero-day vulnerabilities using different methods.. The objective was to enhance the precision and speed of detection through these techniques. The authors [7] conducted experiments on a dataset of acknowledged vulnerabilities and evaluated the performance of 34 various machine and deep learning classifiers. The results indicated that the random forest classifier had the greatest accuracy. To find the best deep-learning model for assessing network security, the authors [8] aim to evaluate multiple deep learning frameworks on diverse network datasets. They want to demonstrate that these models are capable of continuously improving intrusion detection systems and that deep learning enables the accurate real-time detection of cyberattacks. The authors of another research paper [9] aimed to address the challenge posed by zero-day attacks by proposing a new approach for network intrusion detection. Therefore, they proposed using Benford’s law, a mathematical principle used to identify unusual patterns in data, as a new way to extract important features from network traffic data. This will enhance the performance of NID based on machine learning in the detection of zero-day attacks and, through such design, enhance the protection of network systems.

Another author [10] states a method to detect the network intrusion, including zero-day, which is based on the TLBOSA hybrid algorithm. The method enhances IDS’s performance. Here, TLBOSA picks relevant features from data of higher dimensions and tries to eliminate less important ones. The TLBOSA algorithm applies Simulated Annealing into the Teaching Learning Based Optimization method. It used Support Vectors Machines to classify the attacks with good accuracy. The authors investigate the effectiveness of TLBOSA by using two large datasets, such as NSL-KDD and UNSW-NB15. The experimental results demonstrate that it outperforms some other existing algorithms with higher detection rates, accuracy, and false alarm rates. The author claims that this technique can discover zero-day vulnerabilities in the network with appealing IDS performance.

### Traditional methods

The author [11] surveys learning-based WAFs, and estimates how well it could be capable of fighting against zero-day attacks. This susceptibility to zero-day attacks may be addressed by learning-based WAFs, which could also seem much easier to configure and manage. Further work is needed to evaluate how well machine learning-based WAFs can perform in the defense of existing patterns of attacks that target web application frameworks. This review intends to identify the strengths and weaknesses of machine learning-based WAFs and outline open issues that need to be addressed to enhance their performance in protecting web applications against attacks.

The proposed framework [12] aims to detect and rank the severity of zero-day attacks, which are vulnerabilities in seeks to identify and rank zero-day attacks, which are software or application vulnerabilities that attackers can exploit This will enable it to detect unknown network flaws. Testing revealed a 96% rate with a false positive rate as low as 0.3% in a network environment. In any case, the proposed new framework will be a promising candidate for enriching the security of networked applications based on the automatic detection and prioritizing of zero-day attacks. The author [13] reveals a mechanism of zero-day attack detection and prevention through a modified sandbox tool, Cuckoo, for Software-Defined Networks. This mechanism zeroes in on unknown vulnerabilities exploited in zero-day attacks. A look at the design of the system shows effective elimination of the spread of zero-day malware by the isolation of infected clients. Preliminary results suggest that the design may have promise for this proposed methodology with respect to zero-day attack detection and prevention mechanisms for SDN.

The author [14] presents a hybrid three-layer architecture to detect and evaluate the risk of zero-day attacks. The framework aims to integrate with an organization’s defence system to prevent these attacks, which exploit unknown vulnerabilities. The first layer uses statistical, signature, and behavior-based methods to detect unknown vulnerabilities. The second layer assesses the risk level, and the third layer processes information from the first two layers through a centralized database and server. The proposed framework shows an 89% detection rate and a 3% false positive rate in network environment testing.

The author [15] examines the use of machine learning and deep learning for malware detection, focusing on opcode frequency as a feature. The study found that the Random Forest algorithm was more effective than Deep Neural Networks and that basic functions like Variance Threshold outperformed Deep Auto-Encoders for feature reduction. The work highlights the difficulties and open questions in malware detection, identifying the limitations and potential future directions for research. The aim is to advance the development of more efficient and effective ways to detect and defend against malware. The author [16] discusses the challenges of using machine learning for detecting malware in real-world scenarios. The author argues that dynamic malware analysis will be crucial for the success of antimalware systems. The paper presents a review of machine learning for malware detection and identifies three key challenges that limit its effectiveness. Solutions and requirements for future malware detection systems are also discussed. The goal is to highlight the challenges and potential directions for using machine learning in malware detection.

In this work, the author [17] proposes a framework for malware detection using dynamic analysis and machine learning. The Cuckoo sandbox is used for executing malware in an isolated environment and generating a report on system activities during execution. The framework includes a feature extraction and selection module to identify important features for high accuracy and efficient computation. The approach shows improved detection and classification accuracy compared to current methods, offering a promising solution for improving malware detection. Android malware is becoming common as Android applications are increasingly used in the mobile environment [18]. Although prior studies have shown that machine learning is a viable method for identifying Android malware, there is still more to be discovered in this field. Background data on Android apps, including the Android system architecture, security measures, and categorization of Android malware, are provided by the author. The present status of research on machine learning-based Android malware detection is then examined and described, encompassing crucial elements such as sample gathering, data preprocessing, feature selection, machine learning models, algorithms, and the assessment of detection efficacy.

The author [19] proposes a new model, DWOML-RWD, for detecting and preventing ransomware attacks. Ransomware is a major threat to computer systems and requires prompt attention. The DWOML-RWD model combines enhanced krill herd optimization (EKHO), dynamic oppositional-based learning (QOBL), dwarf mongoose optimization (DWO), and an extreme learning machine (ELM) to achieve effective feature selection, classification of good ware and ransomware, and optimal parameter selection in ransomware detection. This model has provided an encouraging method of ransomware detection and prevention in the IoT system.

### Cybersecurity through reinforcement learning

The authors [20] suggest an entirely new approach to the optimization of IDS performance using DRL algorithms. Instead of a traditional environment, they use the sampling function of recorded intrusions as a pseudo-environment to generate rewards based on detection errors during training. This method is tested on the NSL-KDD and AWID datasets and outperforms other machine learning models in terms of performance in intrusion detection and faster classification. It further compares results achieved with other machine learning models and gives insights from different DRL model designs. The authors [21] investigate the application of reinforcement learning (RL) in cybersecurity to increase resilience against various vulnerabilities. They develop the Cyber-Resilient Mechanism (CRM) using RL algorithms that can adapt in real time to threats and uncertainties. The paper also addresses potential weaknesses of RL algorithms and provides defense mechanisms, discussing future challenges and potential uses of RL in cybersecurity and resilience.

A survey paper [22] on using deep reinforcement learning (DRL) in cybersecurity examines existing research on using DRL for Internet protection, intrusion detection, and defense strategies against cyber-attacks through game theory simulations. The paper provides insights and future directions on the potential of DRL in tackling cybersecurity challenges. The authors [23] discuss the use of deep reinforcement learning techniques for cybersecurity threat identification and defense. They review how these methods have been applied in various ways in the cybersecurity field and show promise for enhancing systems with capabilities of general artificial intelligence. The paper aims to offer a thorough analysis of deep reinforcement learning’s applications in cybersecurity, particularly in threat detection and endpoint security. The authors [24] propose a paradigm for utilizing reinforcement learning to enhance decision-making in cyber protection, particularly for defending against online assaults on internal networks. They address the difficulties and advantages of utilizing reinforcement learning at each of the four crucial stages in the threat lifecycle: pentest, design, reaction, and recovery. The authors also review previous studies in this field and suggest potential lines of investigation for further research.

The authors [25] propose a cybersecurity evaluation method for electric power systems (EPS) that combine renewable energy sources (RES) using deep reinforcement learning (DRL) and the Common Vulnerability Scoring System (CVSS). The approach considers sporadic RES generation, vulnerabilities induced by technological devices, and the findings of contingency analysis. The CVSS score is modified using the DRL technique, and the best attack transition strategy for EPS is identified using N-2 contingency findings. Numerical and real-time simulation tests show the method’s scalability and performance in discovering the best attack strategy without requiring complete system observation. The authors in reference [26] discuss a survey of the application of machine learning to zero-day attack prevention. This is indeed a vital threat that has been arising in computer security. In this regard, different models of machine learning, different testing and training datasets, and findings from previous assessments are compared. Future research in this area is in order; it highlights the challenges associated with the application of machine learning to detect zero-day threats along with possible future directions for this type of study.

In [27] the authors provide an automatic vulnerability analysis (AutoVAS) for software vulnerability forecasts through a deep learning system. The source code embedding vectors are created, and they leverage data obtained from the National Vulnerability Database (NVD) and the Software Assurance Reference Database (SARD). The performance is much better compared to that of prior methods with low false negative rates and false positive rates upon testing. Experimenting AutoVAS with Nine Open-Source Projects The approach has been tested on nine open-source projects and found eleven vulnerabilities. The authors [28], describe HMM TDL as a deep learning model aimed at the detection and prevention of zero-day attacks on the cloud platform. The three phases of the model differ: the first uses the Hidden Markov Model (HMM) to detect attacks and forward hyperalert for prevention into a database; the second applies transudative deep learning with k-medoids clustering for attack detection and provides soft labels to attack data, updating that in the database; finally, it updates the database based on a calculated value representing trust when preventing attacks in the cloudThe authors [29] have presented a zero-shot learning method to critically evaluate performance in zero-day attack detection using machine learning models in network intrusion detection systems. They mapped features in network data to known classes of attacks and then ascertained the capability of models to classify unseen, zero-day classes. They propose a new metric, the Zero-day Detection Rate, to measure the quality of the model in detecting such attacks. The results show that, except for a small number of attack classes, most classes are of little concern and each group has its own features’ distribution differing from other attacks. This helps in understanding the capability of machine learning-based models to detect zero-day attacks in NIDS.

The authors [33] suggested the opportunity to apply machine and deep learning for the detection of zero-day attacks within the framework of intrusion detection systems. Their solution was the comparison of performances autoencoders and one-class support vector machines on two IDS datasets. In comparison, results show that autoencoders have high accuracy in detecting zero-days up to 75–99%. According to the research results, autoencoders may be a promising approach to detection of zero-day attacks in IDS. The authors [30] study the use of machine learning and deep learning to detect zero-day attacks in intrusion detection systems. They compare the performance of autoencoders and one-class support vector machines on two IDS datasets. The results show that autoencoders have high zero-day detection accuracy, ranging from 75–99%. The study suggests that autoencoders may be a promising approach for zero-day attack detection in IDS.

The authors [31] look towards the adoption of machine learning and deep learning in zero-day threat detection in intrusion detection and prevention systems. In this regard, they outline the different research on the paper at hand and compare the outcomes against several algorithms used with respect to zero-day attack spotting. The authors describe the difficulty of detecting zero-day attacks by the above approaches and outline the necessity of further work in this direction to understand the problems better and overcome them. Deep Neural Networks (DNNs), which are susceptible to adversarial instances, present a risk to the privacy maintained on these devices. A family of selective gradient sign iterative algorithms has been developed as a solution to this problem, making adversarial instances helpful in preserving the privacy of images stored in IoT devices. This technique can be applied to a large number of images stored on the device and reduces visual distortions without compromising time effectiveness [32,33].

In criminal investigations, the rise of electronic gadgets has led to growing interest in the application of digital forensics. However, traditional methods are limited when the suspect’s disk is encrypted. To overcome this challenge, a new approach has been proposed that involves acquiring forensic data from RAM when the computer is shut down. The findings show that the RAM chips exhibit some level of remanence, allowing investigators to access crucial information that may have previously been lost. The recovered data could help to detect and classify malware [34,35], because this recovered information might contain traces of malware [36,37]. Security and privacy are prime issues, and CBAS based on the certificate is a prevalent means to remedy these problems in the Industrial Internet of Things (IIoT). However, the previous CBAS schemes are not secure and efficient. To overcome these limitations, a new construction of the CBAS scheme is proposed with improved security and efficiency. Based on the discrete logarithm problem hardness, we prove the security of this new scheme in the random oracle model. Security and privacy of the IIoT system and of malware detection and classification process are crucial [38].

Public key encryption with keyword search (PEKS) has recently gained attention, but its security is weak against outsider attacks, and it has high ciphertext complexity. To overcome such issues, they proposed a new framework of identity-based broadcast encryption with keyword search against insider attacks (IBEKS-IA) [39]. This framework allows secure data retrieval for multiple receivers from the attacker and is resistant to insider attacks. One application of these secure data sharing and retrieval methods is in the analysis and identification of malicious software, as securely storing and sharing data is critical to distinguish and evaluate malicious software [40].

## Materials and methods

### Zero-day vulnerability identification framework

The approach and methods used in the study, as well as the methodological framework are outlined. In this work, we demonstrate how Reinforcement Learning can be used to identify zero day vulnerabilities by training a DQN agent to recognize pattern and features in data indicative of zero-day vulnerability. Using a dataset of malwares and benignly samples, the DQN feeds on the dataset and learns to make a classification based on the features it learned. Once training is done, the DQN agent can identify new unseen data points as either malicious or benign. In fact, let’s denote the set of malware and benign samples as D. The DQN agent f(x) will be represented by a function, x is a sample from the D. The agent’s goal is to maximize the accuracy of its predictions about the class label (malicious or benign) of a sample x, denotedy. This accuracy can be represented by a reward signal, denoted by R(f(x),y). The DQN agent learns from the dataset D by updating its parameters, denoted by θ, through an optimization process, such as gradient descent, to minimize the difference between its predictions and the true class labels, as represented in Eq. (2):


$$\theta =\mathrm{\backslash arg}\min_{\theta }[R(f(x;\theta ),y)]$$ (2)

Once the DQN agent has been trained, it can be used to make predictions on new, unseen data points x’ by applying the learned function $f(x^{\prime };\theta )$.

To train the DQN agent, we use a combination of supervised learning and reinforcement learning. During the supervised learning phase, the DQN agent is provided with a large dataset of labeled samples (malware and benign) and it learns to classify these samples based on the features it has learned. The DQN agent is then tested on a separate, unseen dataset and its performance is measured.

Let’s denote the supervised learning dataset $D_{supervised}$ and the reinforcement learning dataset $D_{RL}$. The class label of a sample x is denoted by y.During supervised learning, the DQN agent learns to minimize the loss L between its predictions and the true class labels as shown in Eq (3):


$$L=\frac{1}{\left|D_{supervised}\right|}\sum_{x\in D_{supervised}}Loss(f(x;\theta ),y)$$ (3)

where Loss isachosenlossfunction,suchascross−entropyloss,and θ represents the parameters of the DQN agent. During the reinforcement learning phase, the DQN agent is provided with a stream of data points and must classify each data point as either malicious or benign. If the DQN agent makes an accurate prediction, it is rewarded. If it makes an incorrect prediction, it is punished. The DQN agent learns from its mistakes and adjusts its classification strategy to maximize the reward signal. During reinforcement learning, as shown in Eq (4), the DQN agent learns to maximize the expected reward signal R:


$$R=E[R(f(x;\theta ),y)|x\in D_{RL}]$$ (4)

**Fig 1:**
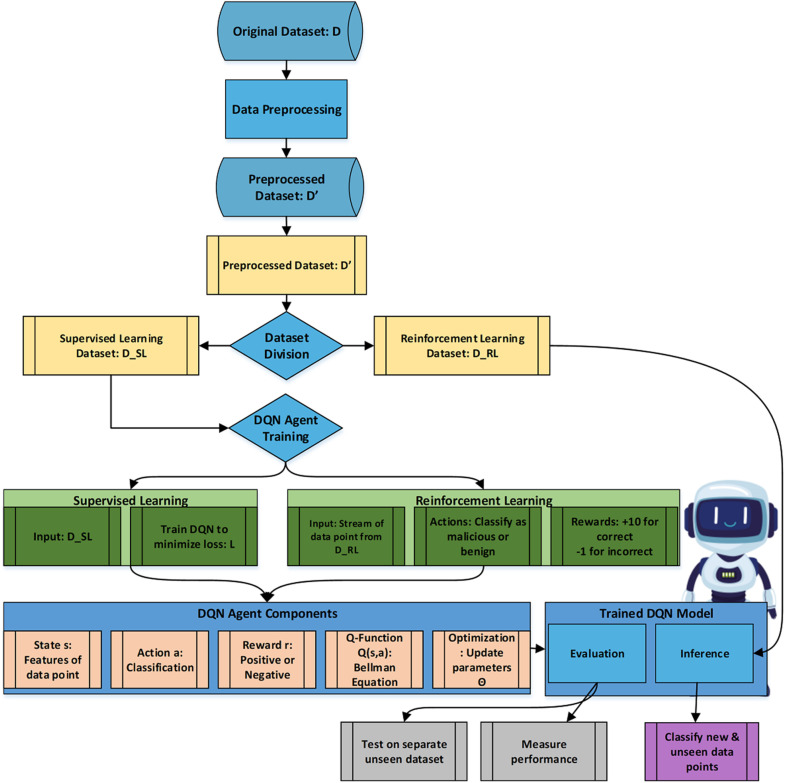
A methodology for Zero-Day Vulnerability Identification.

Where R(f(x;θ),y) is the reward signal, which measures the accuracy of the DQN agent’s predictions. The DQN agent updates its parameters θ through the optimization process, such as Q-learning or SARSA, to maximize the expected reward signal as shown in Fig 1.

### DQN agent

In our approach, we use a DQN agent as the reinforcement learning agent to identify zero-day vulnerabilities. DQN is a type of reinforcement learning algorithm that employs a neural network to learn from data and make decisions. We taught the agent to act in a way that maximized a reward signal, which could potentially have been the best ratio of acting available to it. Here, we considered the accuracy of its predictions with respect to the zero-day vulnerabilities present in the data. In this case, the DQN agent was trained on file sets of malware and benign samples to learn how those patterns and features help the data discover zero-day vulnerabilities. Well after training the DQN agent, it can now tell if new data points (which it has not seen before) is malevolent or benign. It learns the action against the features discovered, with the learned decision making the class label of the data points. The agent of the DQN is then awarded to the performance when they make the right predictions now, so it also learns what it is getting wrong so that the agent can improve over time.

Let’s denote the state of the environment as $s_{t}$, the action taken by the agent at state $s_{t}$ as $a_{t}$, and the reward received by the agent after taking action $a_{t}$ as $r_{t+1}$. The goal of the DQN agent is to learn a function $Q^{*}(s,a)$ that estimates the maximum expected reward when taking action a at the state s and then following the optimal policy thereafter.

The DQN agent is trained using a deep neural network, with the weights and biases of the network denoted by θ. The agent updates its estimates $Q^{*}$ over time by minimizing the loss function L(θ), which is defined as the mean squared error between the estimated Q values and the target Q values.

The target Q values are calculated using the Bellman equation as shown in Eq (5):


$$Q^{\left(s_{t},a_{t}\right)}=r_{t+1}+\gamma \max_{a}Q^{\left(s_{t+1},a\right)}$$ (5)

Where γ is the discount factor, which establishes the significance of future benefits while determining aims.

The DQN agent updates its weights and biases θ using backpropagation and an optimizer, such as Adam, with a learning rate specified by the “critic optimizer learning rate” hyperparameter. L2 regularization is applied by adding a penalty term to the loss function proportional to the sum of the squares of the weights and biases, with the strength of the regularization controlled by the “L2 regularization” hyperparameter. The targets are smoothed by averaging them with the current estimates of the action-value function, controlled by the “target smooth factor” hyperparameter, to stabilize the training process. If the “double Q-learning” hyperparameter is set to “Yes,” the current estimates of the action-value function are used to select the best action rather than the targets, reducing the overestimation of action values.

The replay buffer of collected experiences is either reset or saved, as controlled by the “reset buffer” and “save buffer” hyperparameters, and the target action-value function is updated with a frequency specified by the “target update frequency” hyperparameter. The gradients of the loss function are clipped if they exceed a certain value, controlled by the “gradient threshold” hyperparameter, and prevented from division by zero using the “denomination factor” hyperparameter. Additionally, the gradients of the loss function can be decayed over time using the “gradient decay” and “squared gradient decay” hyperparameters to prevent getting stuck in local minima.

The process is repeated until the DQN has converged, meaning the loss has stopped decreasing and the action-value function has reached a stable state.

### Preprocessing and feature selection

Preprocessing refers to the steps taken to prepare a dataset for analysis or modeling. Before training the DQN agent, we performed several preprocessing steps on the dataset. The dataset consisted of 56 features, and we dropped 2 of these features because they were not deemed important for the classification task. We then divided the remaining 54 features into two sets: a set of numerical features and a set of categorical features. For the numerical features, we performed standardization, which involved scaling the data to have zero mean and unit variance. This helps to ensure that the data is on the same scale and does not dominate the model due to large-scale differences. After preprocessing, the dataset was ready for training the DQN agent.

Let’s denote the original dataset as D, and the preprocessed dataset as D’. The set of numerical features is denoted by N, and the set of categorical features is denoted by C. The number of features in the original dataset is denoted by n. The preprocessing steps can be represented mathematically as follows:

Removing 2 features: n=n−2

Standardization of numerical features as shown in Eq (6):


$$N_{standardized}=\left(\frac{N-mean(N)}{std(N)}\right)$$ (6)

Concatenation of numerical and categorical features as shown in Eq (7):


$$D^{\prime }=[N_{standardized},C]$$ (7)

where mean and std denote the mean and standard deviation, respectively.

### Preparing RL Environment and Agents

Let’s denote the observations as states s, and the class label as y. The action taken by the agent is represented as a, and the reward received by the agent is represented as r.

The agent can take two actions, represented by a∈−1,1. If the agent takes action a=−1, and the true label is y=−1, then the reward received is positive, r=10. If the action taken by the agent is incorrect, i.e., a=−1 and y=1, or a=1 and y=−1, then the reward received is negative, r=−1. The objective of the RL environment and agents is to learn a policy π(s) that maximizes the expected cumulative reward over a given episode. We have 54 features as input to your reinforcement learning (RL) agent and 2 class labels, we can use these features to train the RL agent to make predictions about the class labels. This is known as a supervised learning task, where the goal is to train the RL agent to map the input features to the corresponding class labels. A mathematically described equation, the Bellman equation, is used in reinforcement learning which relates the value of a particular state to those of its successor states. It is used to estimate the expected reward we are going to get from any state by performing the certain action and helps the RL agent learn to search efficiently while following RL.‘It is this equation that Richard Bellman named after him which defines the Q function on which the RL agent’s actions response and select actions achieving maximum reward. This is very basic thing in RL and for most RL algorithms important.

### Preparing dataset for reinforcement learning environment

For reinforcement learning to be used in finding zero-day vulnerabilities, we will need to map data into a form that the RL agent can consume easily. It’s a multi-step process: you break down your data, which is now in training and test sets, and you define what makes an Agent’s state and action space and how you want the Agent to evaluate its performance as a reward function. Over the training set, the RL agent will be trained and over the testing set, we will evaluate. This is important because if we do not divide this data, then the RL agent may have seen the testing data in training, and there is no way for us (in a fair way) to evaluate the RL agent’s performance. The state space is that of information given to the RL agent at each step of the decision-making process. In our case, the features being used to make decisions about the data point being classified by the RL agent form our state space. The action space, which is set of actions the RL agent can choose to do at each step, is the same. In our case, the action space is the two class labels (malicious or benign) to which any RL agent can assign a data point.

Let the original dataset be denoted by D, the training set $D_{train}$ and the testing set $D_{test}$. The division can be represented as shown in Eq (8):


$$D_{train}\cup D_{test}=D\;and\;D_{train}\cap D_{test}=\emptyset$$ (8)

The state space and action space can be defined as shown in Eq (9):


$$S=s_{1},s_{2},...,s_{n}$$ (9)

Where $s_{i}$ is the feature vector of the $i^{th}$ data point in the dataset.


$$A=a_{1},a_{2}$$ (10)

Where $a_{1}$ and $a_{2}$ represent the two class labels (malicious or benign) as shown in Eq (10).

The reward function, which evaluates the agent’s performance, can be represented as shown in Eq (11):


$$R(s,a)=\{\begin{array}{cc} {}*20c0 & if\;a=groundTruth\\ -1 & otherwise \end{array}$$ (11)

Where $ground_{t}ruth$ is the actual class label of the data point, and R(s,a) is the reward for taking action a in the state s.

The reward function is a key component of the RL environment, as it defines the goal of the RL agent and determines how it will be evaluated. In our case, the reward function is designed to encourage the RL agent to make accurate predictions about the class labels of data points. The RL agent will receive a positive reward for making correct predictions and a negative reward for making incorrect predictions. By learning to maximize this reward signal, the RL agent will learn to classify data points accurately. An RL (reinforcement learning) environment is a simulated or real-world system in which an RL agent (such as a DQN agent) can interact and make decisions to achieve a specific goal.

The reward function can be mathematically defined as follows:

Let A be the action taken by the RL agent and $A_{actual}$ be the actual class label of the data point. Then, the reward R can be given as represented in Eq (12):


$$R=\{\begin{array}{cc} {}*20l10 & ifA=A_{actual}\\ -1 & otherwise \end{array}$$ (12)

Where the reward of 10 is for a correct prediction and the reward of −1 is for an incorrect prediction. The goal of the RL agent is to maximize the cumulative reward over a sequence of predictions by making accurate classifications.

An RL environment consists of several components. The state space represents the information that the RL agent has access to at each step of the decision-making process. In the context of identifying zero-day vulnerabilities using a DQN agent, the state space might consist of the features of the data point being classified. The action space represents the choices the RL agent can make at each step. For zero-day vulnerability identification, this might include the two class labels (malicious or benign) that the DQN agent can assign to a data point. The reward function is crucial as it defines the goal of the RL agent and determines its evaluation criteria. In this context, the reward function encourages the DQN agent to make accurate predictions about data point class labels, with positive rewards for correct predictions and negative rewards for incorrect ones. The transition function defines how the state of the environment changes due to the RL agent’s actions, such as how the data point being classified changes with the DQN agent’s decision. The termination condition is finally specified as when to stop the interaction of the RL agent with the environment - perhaps after the DQN agent had verified the number of data points/the level of accuracy.

The termination condition is finally specified as when to stop the interaction of the RL agent with the environment - perhaps after the DQN agent had verified the number of data points/the level of accuracy. If we are clear about the state space, action space, reward function, transition function and termination condition then let be the set of states, be the set of actions, be the reward function, be the transition function and be the terminating condition. We can define as an RL environment. In other words, the state space stands for the information which the RL agent possesses when it makes his choice at each step of the decision-making process. The choices of the RL agent are also defined with the action space. The reward function describes how the RL agent would be scored and how it would attain a goal. RL agent’s actions with regard to the environment state changes are defined by the transition function. In the case of the RL agent interaction with the environment, the termination condition sets out when the interaction should stop, which might be after the DQN agent has classified a certain number of data points or achieved a specific level of accuracy.

Let S be the state space, A be the action space, R be the reward function, T be the transition function, and $T_{c}$ be the termination condition. An RL environment can be defined as $\left(S,A,R,T,T_{c}\right)$. The state space S represents the information available to the RL agent at each step of the decision-making process. The action space A represents the choices the RL agent can make. The reward function R defines the goal of the RL agent and how it will be evaluated. The transition function T defines how the state of the environment changes as a result of the RL agent’s actions. The termination condition $T_{c}$ defines when the RL agent’s interaction with the environment should end. Then we can define these components (environmental components) of the RL environment so we can build a simulated or real-world system for the DQN agent to interact and make decisions over, aiming to achieve a goal, i.e., correctly classifying data points as malicious or benign.

### Working of DQN agent

An RL (reinforcement learning) agent which approximates some Q function or Q function is called a DQN (Deep Q Network) agent. This approximated Q function is then used by the DQN Agent to choose actions which are hoped to maximize the reward signal in the environment. The RL environment used by the DQN agent works in the following manner: Initialize the agent, begin collecting observations and experiences, train the agent with collected experiences, select actions using approximate Q function and observe resulting state and reward. The optimal action-value function is approximated using a neural network and actions are chosen via the max covariance search (MCS) that maximizes the reward signal by the DQN.

Let S be the state space, A be the action space, R(s,a) be the reward function, and $T(s,a,s^{\prime })$ be the transition function. Let $Q^{*}(s,a)$ be the optimal action-value function.

A DQN agent, denoted as DQN, can be represented as:

Initialize DQN with parameters θ.

Collect observations and experiences $\left(s_{t},a_{t},r_{t+1},s_{t+1}\right)$ at each time step t by taking action $a_{t}$ in state $s_{t}$, obtaining a reward $r_{t+1}$, and transitioning to state $s_{t+1}$. Store the experiences in a replay buffer.

Train DQN by updating its parameters θ to minimize the loss function L(θ): $L(\theta )=𝔼(s,a,r,s^{\prime })\sim U(D)[{\left(r+\gamma \max a^{\prime }Q(s^{\prime },a^{\prime };\theta^{-})-Q(s,a;\theta )\right)}^{2}]$ where U(D) is a uniform sampling from the replay buffer D and $\theta^{-}$ are the target network parameters.

Select actions in the state s by choosing $a=\mathrm{\backslash arg}\max_{a^{\prime }}Q(s,a^{\prime };\theta )$.

Observe the resulting state s’ and reward r’ and repeat from step 2.

### Data input to DQN agent

The method of data input for the RL (reinforcement learning) agent will depend on the specific details of the environment and the learning algorithm used. Here are a few common methods of data input that could be used in the context of an RL agent for identifying zero-day vulnerabilities: The RL agent could be provided with raw data points, such as the features of a data point being classified as malicious or benign.

In such case, the RL agent will be responsible for processing and extraction of the relevant information from the raw data and predict accordingly. Instead, our RL agent could be fed (preprocessed) data points like numerical features normalized or categorical features encoded in one hot. It can relieve some burden on the RL agent and the data can be learnt more easily. Data augmentation is simply generating new, synthetic data points from the current data to help increase the size and data diversity of the dataset. It can be useful in helping improve the performance of the RL agent and make it more robust to changes in the environment. The specifics of the environment together with a learning algorithm used, resource availability and the given constraints all make the most appropriate method of data input specific to the RL agent.

One technique to improve generalization performance of an RL (reinforcement learning) agent is randomization of the data. By breaking the data up, shuffling it and exposing it to the agent in random order, there is a chance to lessen the possibility for the agent to over fit the training data, and instead, increase its ability to learn from un seen data. Nevertheless, it’s worth saying that randomizing its data is not always the best thing to do. The data might also present in some natural ordering structure or sort that the RL agent needs to learn from. To illustrate, randomizing our data is an example when the data is a sequence of events and randomization might not only wipe out the temporal dependencies of the events, but it could be more difficult for the RL agent to learn from the data. And given the characteristics of the data and the learning algorithm, the decision to randomize the data will typically depend both an overall and on specifics of the environment. In general, this is a good idea because you want to try various data orders and benchmark the RL agent in order to determine what is the best way to go.

Let the data be represented by D, and let the order of the data be represented by O. Randomizing the data can be done by shuffling O, resulting in a randomized order of the data O’. The generalization performance of the RL agent can be represented by $P_{gen}$. The effect of randomizing the data on the generalization performance can be expressed as:

$P_{gen}(O^{\prime })=f(D,O^{\prime })$, where f represents the function that maps the data and the order to the generalization performance of the RL agent. It is important to note that f may not always be the same for different data and order combinations. In some cases, preserving the natural ordering or structure of the data may be important for the RL agent to learn, resulting as shown in Eq (13):


$$P_{gen}(O)=f(D,O)>P_{gen}(O^{\prime })$$ (13)

In general, the best approach for the data order can be determined by evaluating the performance of the RL agent for different data orders, expressed as shown in Eq (14):


$$O^{*}=\mathrm{\backslash arg}\max_{O}P_{gen}(O)$$ (14)

Where $O^{*}$ represents the optimal order of the data that maximizes the generalization performance of the RL agent.

## Results and discussion

The following is a research work reporting results in newly developed techniques to be used in the identification of zero-day vulnerabilities, based on reinforcement learning. This paper assesses the efficacy of using reinforcement learning in the identiﬁcation of zero-day vulnerabilities, or, equivalently, of the vulnerabilities that are yet to be discovered or disclosed. It used a DQN agent to classify data points as either malicious or benign, given some features and then proceeded to collect a variety of metrics measuring the performance of the DQN agent. Findings shed light on the capabilities and limitations of applying reinforcement learning to zero-day vulnerability identification for further approaches in the area. Overall, this work proposed an analysis of how well a method of reinforced learning could be used in finding zero-day vulnerabilities and contributes to existing knowledge in cybersecurity literature.

### Hyper parameters selection

Hyperparameters give the values applied in the RL agent during the identification of zero-day vulnerabilities. Hyperparameters refer to variables that are set before learning a machine learning model; these variables affect two aspects: learning and the performance of the model. In this case, the sample time refers to the interval at which observations are collected from the environment. The discount factor is a parameter that determines the relative importance of future rewards in the RL agent’s decision-making process. The batch size specifies the number of observations used to update the agent’s knowledge at each training step. The experience buffer length indicates the maximum number of observations stored in the replay buffer.

The hyperparameters in the RL agent can be expressed mathematically as follows: Sample Time, $T_{s}$: The sample time defines the frequency at which observations are collected from the environment and fed into the agent. It is represented mathematically as $T_{s}$. Discount Factor, γ: The discount factor is a scalar value that determines the relative importance of future rewards in the agent’s decision-making process. It is represented mathematically as γ, where 0≤γ≤1. Batch Size, N: The batch size determines the number of observations that are used to update the agent’s knowledge at each training step. It is represented mathematically as N, where N is a positive integer. Experience Buffer Length, L: The experience buffer length defines the maximum number of observations that are stored in the replay buffer. It is represented mathematically as L, where L is a positive integer.

These are hyperparameters for a reinforcement learning agent, specifically a DQN agent. Hyperparameters are values that are set before training a machine learning model and are not learned during the training process. They can have a significant impact on the performance of the model and must be carefully chosen to achieve good results.

A brief explanation of each of the hyperparameters listed. Sample time is the frequency at which the agent samples the environment to gather experiences and update its internal models. A lower sample time may allow the agent to learn faster, but it may also be more computationally expensive. The gradient threshold method determines the method used to threshold gradients during training. The “global-I2norm” method scales gradients so that the sum of their squares is equal to a global value, which can help stabilize training. Experience buffer length is the maximum number of experiences (state-action-reward-next state tuples) that the agent stores in its experience buffer. The experience buffer is used to sample mini-batches of experiences to train the agent’s neural network. Target smooth factor determines how quickly the agent’s target neural network (which is used to calculate the expected value of each action) is updated. A smaller value results in slower updates and may lead to more stable learning. L2 regularization is a type of regularization that is used to prevent overfitting by adding a penalty to the loss function based on the L2 norm of the model’s weights. Discount factor determines the importance of future rewards compared to immediate rewards. A higher discount factor means that the agent places more emphasis on long-term rewards. Critic optimizers learn rate is the learning rate used for the optimizer that updates the critic network, which is used to estimate the value of each action. Double Q-learning determines whether the agent uses the double Q-learning technique, which helps reduce overestimations of action values. Batch size is the number of experiences sampled from the experience buffer at each training step. A larger batch size may lead to more stable learning, but it may also be more computationally expensive. Reset buffer determines whether the experience buffer is reset after each training epoch. Target update frequency is the frequency at which the target neural network is updated. Optimizer is the optimization algorithm used to update the agent’s neural networks. In this case, it is the stochastic gradient descent with momentum (SGDM) optimizer. Save buffer determines whether the experience buffer is saved to disk after each training epoch. Moment is the momentum parameter used for the SGDM optimizer. It determines the amount of momentum applied to the gradients during training as shown in Table 1.

**Table 1:** DQN agent hyperparameters selection at the time of training.

	Hyper Parameter	Values
**1**	Sample time	1
**2**	Gradient threshold method	Global-I2norm
**3**	Experience buffer length	1000
**4**	Target smooth factor	0.001
**5**	L2 regularization	0.0001
**6**	Discount factor	0.98
**7**	Critic optimizers learn rate	0.01
**8**	Double Q-learning	Yes
**9**	Batch size	16
**10**	Reset buffer	No
**11**	Target update frequency	1
**12**	Optimizer	SGDM
**13**	Save buffer	Yes
**14**	Moment	0.9

### Experimental setup

The experimental setup is an Intel(R) Xeon(R) CPU E5-2643 v3 @ 3.40GHz with 32 GB of memory and Windows 10 Pro 22H2 with OS build 19045.2364. The GPU is an Nvidia Quadro M4000 with 8 GB of video memory. The Xeon CPU is a processor commonly used in servers and workstations, known for its multiple cores and high performance and reliability. The Nvidia Quadro M4000 is a professional graphics processing unit (GPU) with a high number of CUDA cores and substantial memory capacity, making it well-suited for tasks such as machine learning and scientific computing. Hardware and software should relate to a task for which an experiment will be applied. In this case, the hardware appears to be well-suited for machine learning tasks, especially if the GPU is utilized to accelerate training. The specific version of Windows and build number may also be important for ensuring compatibility with any software being used.

**Fig 2:**
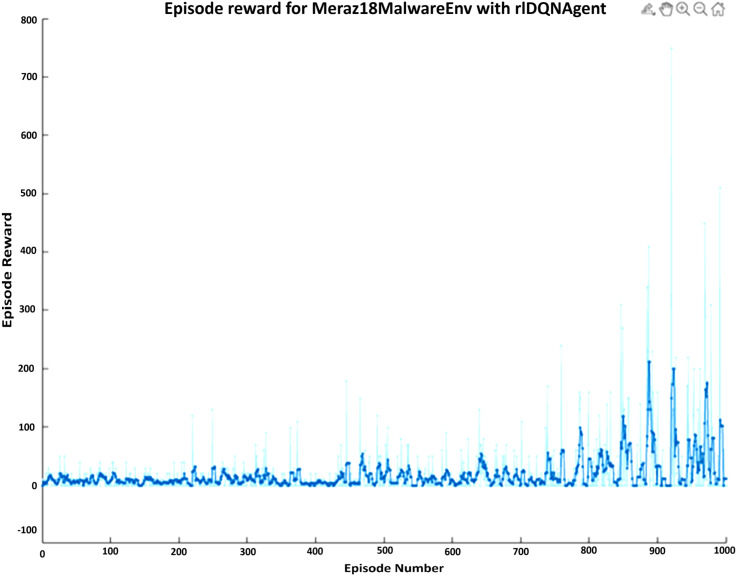
DQN agent training log with 1000 episodes and episode reward.

The training log displays the episode reward for each episode of training. The episode reward is the total reward that the agent receives during a single episode of interaction with the environment. It measures how well the agent is doing and could hence be used to track how an agent improves its performance while training. Every episode is represented through an entry on the x-axis while the episode is rewarded by an entry on the y-axis. The entries along the y-axis can be positive or negative, the positive shows that a reward was given during the episode, in contrast, a negative shows no reward was given. The specific values of the rewards (e.g., -1, 9, 19) represent the amount of reward provided during each episode, which may indicate that the agent has learned to take actions resulting in higher rewards over time, as shown in Fig 2.

After 1000 episodes the statistics by which the DQN agent trains show that the total agent reward ended up being 2885, the average reward per episode was 11, and the average number of steps per episode was 2.2. The sum of all re-wards received by the agent over all episodes is merely the total agent reward of 2885. Total reward normalized by the number of episodes equals 11 - this is the average reward per episode. This metric indicates the agent’s average performance throughout training. The average number of steps per episode, which is 2.2, reflects the average number of actions taken by the agent during each episode. This statistic helps to gauge the complexity of the tasks that the agent is learning to solve, as shown in Table 2.

**Table 2:** DQN agent training statistics after completing 1000 episodes.

	Training Tasks	Action
**1**	Status	Training finished
**2**	Episode number	1000
**3**	Episode reward	-1
**4**	Episode steps	1
**5**	Total agent steps	2885
**6**	Average reward	11
**7**	Average steps	2.2
**8**	Averaging window length	5
**9**	Training stopped by	MaxEpisodes
**10**	Training stopped at	Episode 1000

**Fig 3:**
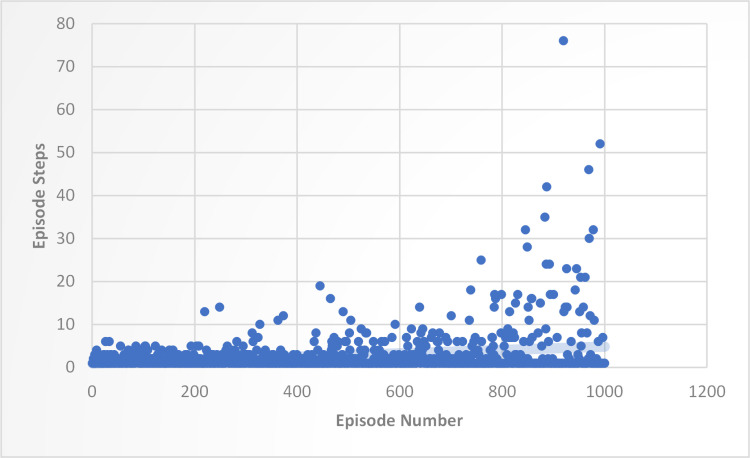
Training Performance of DQN Agent with 1000 Episodes and Episode Steps.

In this experiment, the training statistics tracked over 1000 episodes of the training or learning process. Each episode is represented by an entry on the x-axis, while the number of steps taken during each episode is represented on the y-axis. The y-axis entries vary widely, with some episodes requiring only a few steps and others requiring many more. The average number of steps taken per episode appears to be around 1–6, though there are a few episodes that required significantly more steps, with the maximum number of steps being 76. This variation may indicate that some episodes were more difficult or complex than others, requiring more steps to complete, as shown in Fig 3.

**Fig 4:**
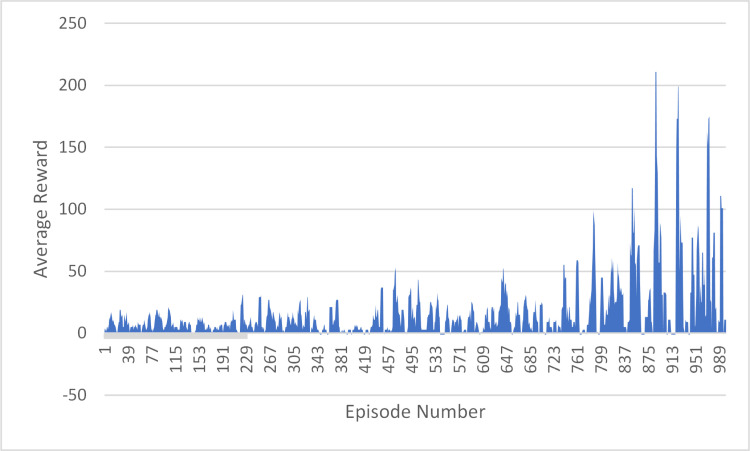
DQN Agent Training Progress: Episode Rewards over 1000 Episodes.

For the first 800 episodes, the number of steps taken per episode appears to be randomly distributed between 1 and 50. However, for the last 200 episodes (from 801 to 1000), the number of steps taken per episode ranges from 1 to 100, with some episodes requiring significantly more steps, up to a maximum of 211. This may indicate that the training process became more difficult or complex as it progressed, requiring more steps to complete each episode. Overall, the training statistics for the model show the number of steps taken during each episode and how this number changed throughout the 1000 episodes, as shown in Fig 4.

**Fig 5:**
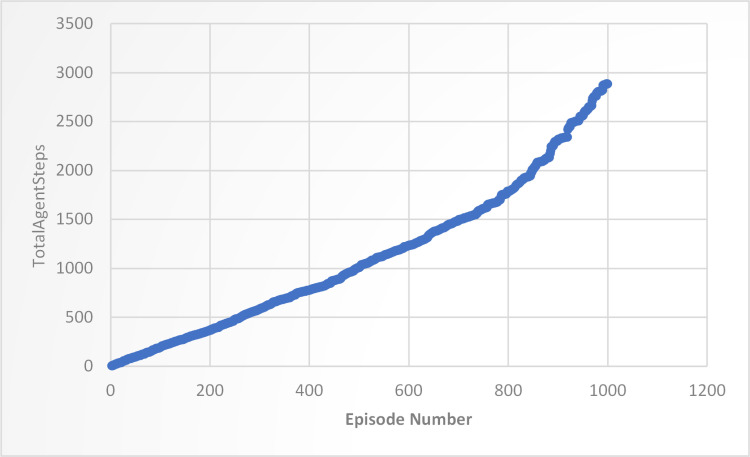
Tracking the performance of a DQN agent over 1000 training episodes.

In this experiment, the trained agent was evaluated over 1000 episodes of training or learning. Each episode was represented on the x-axis, while the cumulative number of steps taken by the agent during each episode was plotted on the y-axis. The y-axis entries show the total number of steps taken by the agent up to that episode. Thus, the first entry on the y-axis represents the total number of steps taken during the first episode, and the last entry represents the total number of steps taken over all 1000 episodes. The total number of steps increased throughout the episodes, starting at 1 and reaching 2885 by the end of the 1000th episode. This increase may indicate that the agent improved its performance or efficiency as it gained more experience through the training process, as shown in Fig 5.

**Fig 6:**
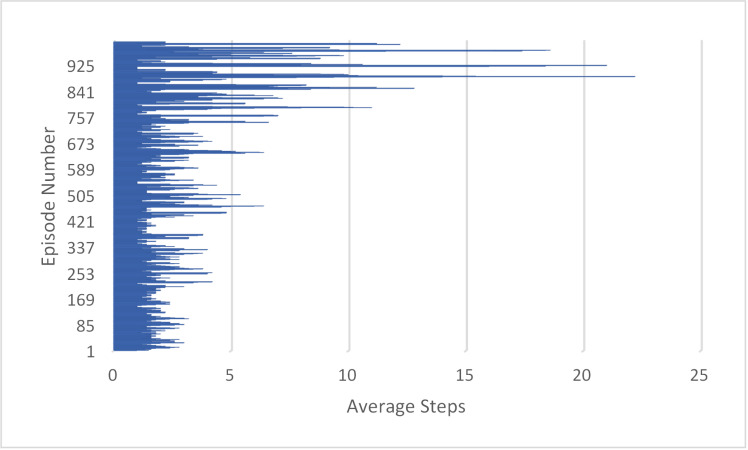
Measuring the Performance of DQN Agent in 1000 Training Episodes and average steps.

In this experiment, the DQN agent was trained through 1000 episodes of reinforcement learning. The training statistics reveal that the total reward received by the agent over these 1000 episodes was 2885. Additionally, the average number of steps per episode ranged from 1.0 to 9.19, with some episodes requiring significantly more steps, up to a maximum of 21. This variation may indicate that some episodes were more difficult or complex, necessitating more steps to complete. The average number of steps per episode provides insight into the overall efficiency of the DQN agent during training and highlights areas where it may be struggling, as shown in Fig 6.

**Fig 7:**
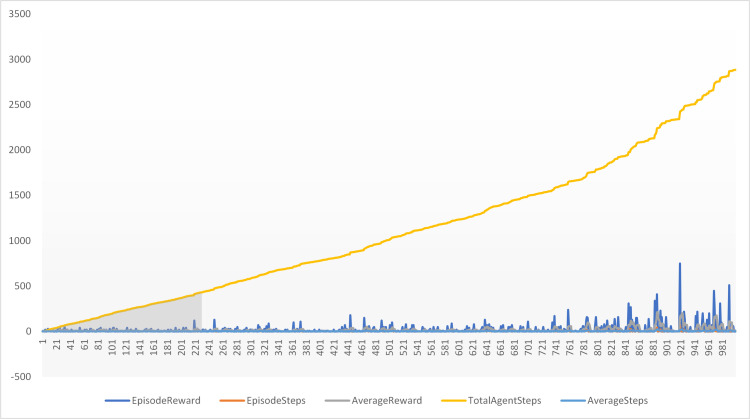
Training coverage of the agent.

**Fig 8:**
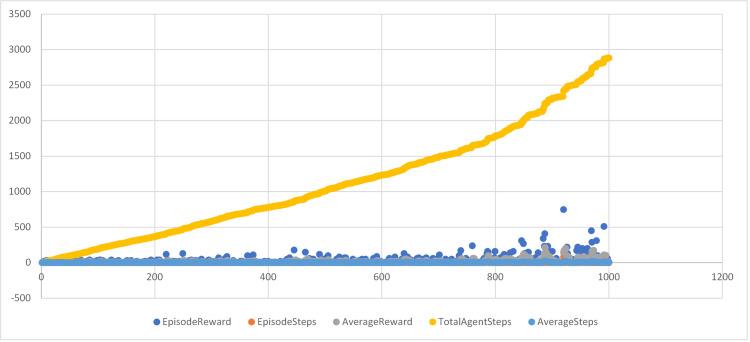
DQN agent Training scatter plot.

The total reward received by the agent after 1000 episodes is 2885, which represents the sum of all rewards collected across the episodes. The average reward per episode is 11, calculated by dividing the total reward by the number of episodes. This figure reflects the agent’s average performance throughout the training process. The average number of steps taken per episode is 2.2, indicating the mean number of actions the agent performed in each episode. This metric helps to gauge the complexity of the tasks the agent is learning to solve. The training log offers a more detailed view of the agent’s performance, including the episode reward, the number of steps taken per episode, the cumulative average reward up to that point, the total number of steps taken, and the cumulative average number of steps per episode. This data is illustrated in Fig 7 and Fig 8.

The training log provides value for each episode of training. The Episode Index column indicates the index of the episode, the Episode Reward column shows the reward received during that episode, and the Episode Steps column displays the number of steps taken by the agent during that episode. The Average Reward column reflects the average reward per episode up to that point in training, while the Total Agent Steps column shows the cumulative number of steps taken by the agent up to that point. The Average Steps column presents the average number of steps taken per episode up to that point in training, as shown in Table 3.

**Table 3:** Training output sample data are taken from the DQN agent.

Episode Index	Episode Reward	Episode Steps	Average Reward	Total Agent Steps	Average Steps
**1**	-1	1	-1	1	1
**2**	9	2	4	3	1.5
**3**	-1	1	2.33	4	1.33
**4**	-1	1	1.5	5	1.25
**996**	-1	1	-1	2875	1
**997**	59	7	11	2882	2.20
**998**	-1	1	11	2883	2.20
**999**	-1	1	11	2884	2.20
**1000**	-1	1	11	2885	2.20

**Fig 9:**
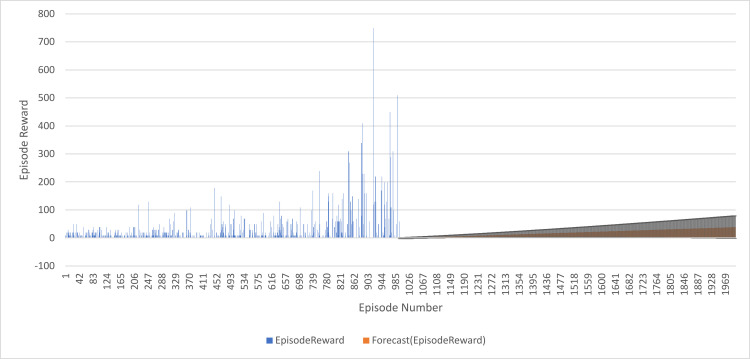
DQN Agent Forecasting Progress: Episode Rewards after forecasted 1000 Episodes.

In this study, a time series forecasting model was developed and trained on the first 1000 episodes of a given dataset (Episodes 1 to 1000). The model was then used to forecast the next 1000 episodes (Episodes 1001 to 2000), and the results were presented in the form of forecasted episode rewards along with a 90% confidence interval. Mean Absolute Scaled Error (MASE) (3.52), Symmetric Mean Absolute Percentage Error (SMAPE) (1.59), Mean Absolute Error (MAE) (63.40), and Root Mean Squared Error (RMSE) (102.19), as shown in Fig 9.

**Fig 10:**
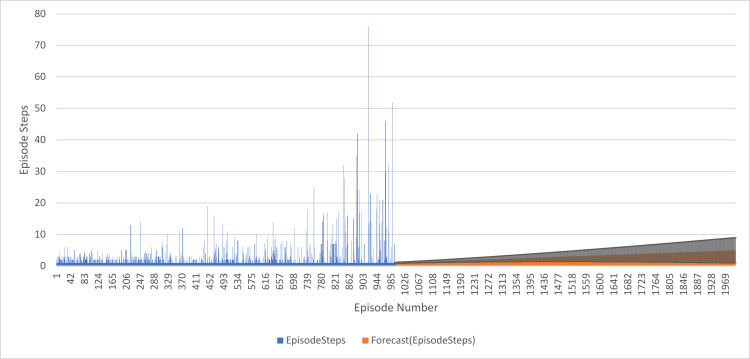
DQN Agent with 1000 training and 1000 forecasted Episode Steps Performance.

The forecast values are close to 1, indicating that the model predicts most episodes will have around 1 EpisodeStep. This aligns with the observation that most episodes in the first 1000 episodes have 1 EpisodeStep. The confidence intervals are very small, which reaches a maximum of 0.33, meaning that the model is very confident about the predictivity of its tool. The values of the forecast increases with the number of episodes and reach a maximum of 6.74. Which means, therefore, that the model predicts EpisodeSteps to increase slowly with time. The plot of the forecasted values shows a nice smooth trend upward in the estimated values, supporting the observation that the model expects gradually increasing EpisodeSteps. These results suggest that the model can adequately make predictions based on the first 1000 episodes and, therefore, can be used to forecast future episode values with a relatively high degree of certainty, as seen in Fig 10.

**Fig 11:**
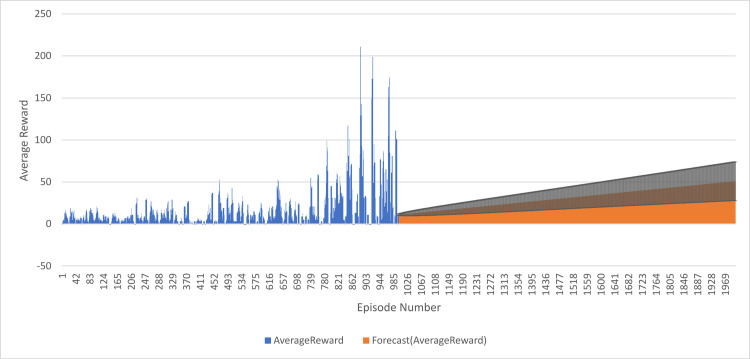
Performance of the DQN Agent: Episode Rewards after Predicted 1000 Episodes.

These average reward values do increase steadily with the episodes. On the other hand, average reward goes from -1 at the beginning (first episode), to 11 in the thousandth episode. According to the expected values it is supposed to start with a mean of eleven in the 1001st episode and will climb to 50 from the 2000th episode. Error bars in the plot represent a confidence interval, an estimation of forecasted values’ degree of uncertainty. The confidence intervals are somewhat low for the first 1000 episodes but otherwise increase with the higher values that are being forecasted. That means, as average reward values grow, the model’s predictions become less certain. The results as a whole show that average reward actually grows with time, and that the model is a good predictor for future values of average reward. This implies positive trends in average reward indicating systematically improving performance of the learning process, as the agent gets increasingly good at more and more appropriate choices for doing in the environment under a wider exposure to it as shown in Fig 11.

**Fig 12:**
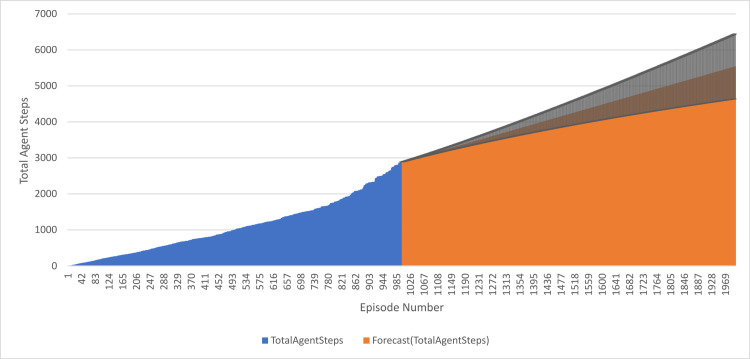
Performance of DQN agent over 1000 training episodes and 1000 forecasted episodes.

The plots above depict how TotalAgentSteps varied with time across 1000 training episodes and 1001–2000 forecasting episodes. The former were periods of training, and after that, the model had produced forecasted values for the latter 1000 episodes. All the values represent a steadily increasing average reward during both periods of training as well as forecasting. It might probably be deduced from the plot that TotalAgentSteps is increasing with time in the case of both periods. Also calculated and printed in the result is the Confidence Interval for TotalAgentSteps. This measures how well the model has performed in the interval it is meant to capture the actual value at a given confidence level. As illustrated above, the Confidence Interval also tends to increase gradually with time. Therefore, this means the more the training data used, the more accurate the better the model becomes is. This result shows that the values of forecasts are well within the training data, and further training adds up to the accuracy of the model. In general, these results express the usability of reinforcement learning models in solving complex problems. The model learned the best strategy for the environment quite well; the trend in both average reward and Confidence Interval shows that the model generalizes its learning to new episodes, as shown in Fig 12.

**Fig 13:**
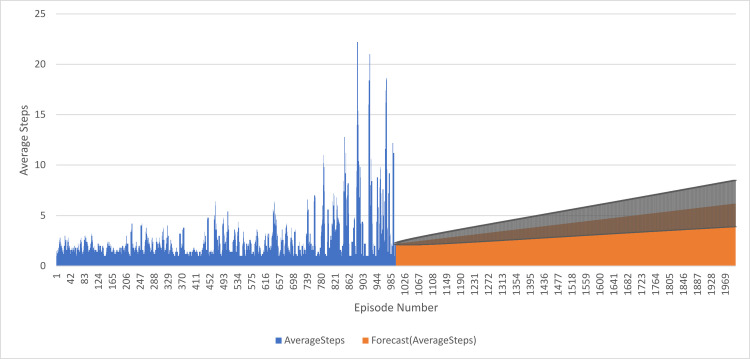
Measure Performance of DQN Agent Predicting Average Steps in 1000 Episodes.

The average reward for the training of the first 1000 episodes is between 1 and 2.5 steps, peaks at 2.7 steps, and at its low point reaches 1 step. Forecasted values: by the end of 2000 episodes average reward continues to creep up steadily to around 3.8 steps. The likelihood is that the agent will continue learning and polishing strategies as it moves through further episodes. A confidence interval contains two values that denote the degree of uncertainty of the forecasted values. The value of the lower confidence interval conveys higher amounts of confidence for the predicted value, and the value of the higher confidence interval conveys less confidence. The confidence interval values are high if the forecasted episodes have values significantly lower than the values from the training episodes which depict how much more confident the model is for the predicted value. More globally, these results mean that the agent improves its strategies step by step, performing fewer steps towards the goal over episodes. Moreover, the predicted values indicate that this trend will continue in the next 1000 episodes, as depicted in Fig 13.

We evaluate the performance of our model comprehensively, used statistical metrics like MASE (Mean Absolute Scaled Error) and SMAPE (Symmetric Mean Absolute Percentage Error) since such metrics are important in dynamic and unpredictable environment such as the cybersecurity environment. MASE scaled the measure of forecast accuracy (forecast error compared to naive benchmark) so that our method outperformed simple heuristics or rulebased systems. As such, the MASE value of 2.78 of TotalAgentSteps indicates that the model runs up to nearly three times better compared to the naive forecasting methods. Likewise, SMAPE provides a symmetric (percentage) measure of error, equally penalizing over and under predictions, while it is important for maintaining consistency in forecasting key parameters, e.g. by attack likelihood or by system anomalies. TotalAgentSteps has an SMAPE value of 0.00, demonstrating perfect precision, and a value of 1.59 for EpisodeReward implies that on average, the average deviation is 1.59%. These metrics combined guarantee that our model is capable to not only effectively detect vulnerabilities but has the generalization ability across different datasets and environments, which poses the core challenge in cybersecurity.

The following table is a statistical evaluation of the forecasting values of the parameters EpisodeReward, EpisodeSteps, AverageReward, TotalAgentSteps, and AverageSteps. The statistical measures used include Alpha, Beta, Gamma, MASE, SMAPE, MAE, and RMSE. Alpha represents the accuracy of the forecast for the EpisodeReward parameter, with a value of 0.10. Beta indicates the bias in the forecast for EpisodeReward, with a value of 0.00. Gamma reflects the precision of the forecast for EpisodeReward, with a value of 0.00. The MASE (Mean Absolute Scaled Error) measures the accuracy of the forecast relative to the average of the actual values. The SMAPE (Symmetric Mean Absolute Percentage Error) measures the percentage error between the actual and forecasted values. For EpisodeReward, the SMAPE value is 1.59, indicating a low percentage error in the forecast. For EpisodeSteps, the SMAPE value is 1.12, indicating a relatively low percentage error in the forecast. The MAE (Mean Absolute Error) measures the average difference between the actual and forecasted values. For EpisodeReward, the MAE value is 63.40, indicating a high average difference between the actual and forecasted values. For EpisodeSteps, the MAE value is 6.34, indicating a relatively low average difference. The RMSE (Root Mean Squared Error) measures the square root of the average of the squared differences between the actual and forecasted values. For EpisodeSteps, the RMSE value is 10.22, indicating a relatively low error in the forecast. The statistical evaluation of the forecasted values provides valuable insight into the accuracy and bias of the forecasts for the different parameters. The results indicate that the forecast for EpisodeSteps has a relatively low error compared to EpisodeReward, as presented in Table 4.

**Table 4:** DQN agent Forecasting statistics after completing 1000 episodes.

Statistic	EpisodeReward	EpisodeSteps	AverageReward	TotalAgentSteps	AverageSteps
**Alpha**	0.10	0.10	0.75	1.00	0.75
**Beta**	0.00	0.00	0.00	0.25	0.00
**Gamma**	0.00	0.00	0.00	0.00	0.00
**MASE**	3.52	3.52	4.70	2.78	4.70
**SMAPE**	1.59	1.12	0.56	0.00	0.33
**MAE**	63.40	6.34	17.74	6.22	1.77
**RMSE**	102.19	10.22	30.39	10.52	3.04

The comparison table outlines various research papers that utilize reinforcement learning (RL) and deep reinforcement learning (DRL) for cybersecurity applications, along with our proposed work on zero-day vulnerability identification. Our paper introduces an RL approach to detect zero-day vulnerabilities in real-time without prior information, demonstrating notable accuracy and precision through statistical measures. Another study applied DRL to intrusion detection, achieving superior results with the Double Deep Q-Network (DDQN) on labeled datasets. Research on RL for cyber resilience highlights the adaptability and strategic response capabilities of RL systems while noting inherent vulnerabilities and future challenges. A review of DRL for cyber-physical systems offers a comprehensive theoretical overview but lacks real-world application data. Another review of DRL applications in threat detection and protection emphasizes innovative and state-of-the-art results yet identifies a gap in comprehensive reviews. A proposed RL framework for network defense focuses on systematic decision-making across different threat lifecycle stages. Lastly, DRL for cybersecurity assessments in wind-integrated power systems showcases effective vulnerability assessment and scalability, though requiring broader real-world validation. This comparison highlights the diverse applications, strengths, and limitations of RL and DRL in enhancing cybersecurity, as shown in Table 5.

**Table 5:** Comparison of Reinforcement Learning Approaches in Cybersecurity Research.

Paper	Focus Area	Approach	Key Results	Strengths	Limitations
**Lip Yee Por et al. (2024) [41]**	AI-Based Detection of Zero-Day Attacks	Systematic Literature Review (SLR)	Identified methods and challenges in zero-day detection	Comprehensive analysis of current methods	Limited coverage of recent methodologies; potential bias in selected studies; reliance on data quality from diverse sources.
**Zhen Dai et al. (2024) [42]**	Intrusion Detection Model	Machine Learning	Developed a model for detecting zero-day attacks	Effective in unseen data detection	Challenges in real-time application
**Lopez-Martin et al. (2020) [20]**	Intrusion detection	Deep Reinforcement Learning (DRL) using NSL-KDD and AWID datasets	Best results with Double Deep Q-Network (DDQN)	Improved intrusion detection performance, faster than alternative models	Requires labeled datasets; manual intrusion identification needed
**Huang et al. (2022) [43]**	Cyber resilience	RL for feedback-enabled cyber resilience	Discusses vulnerabilities and defense methods for RL-enabled systems	Adaptable and strategic responses to known and unknown threats, dynamic response mechanism	RL vulnerabilities, future challenges in securing RL systems
**Nguyen and Reddi (2021) [44]**	Cyber-physical systems security	DRL for cyber defense	Survey of DRL approaches for various cyber defense strategies	Comprehensive review, potential for addressing complex cyber security problems	Lacks real-world application data, theoretical focus
**Sewak et al. (2021) [45]**	Threat detection and protection	Deep RL for cybersecurity	Review of different DRL applications in threat detection	Innovative applications in threat defense, state-of-the-art results	Need for more comprehensive reviews, gap in literature
**Wang et al. (2022) [24]**	Cyber defense decision-making	RL framework for network defense	Defines four modules: pentest, design, response, recovery	Systematic view of decision-making problems, breakthrough in complicated decision-making	Comparison limited to existing research, need for future work identification
**Liu et al. (2020) [25]**	Cybersecurity of wind integrated power systems	DRL for cybersecurity assessment	DRL performs closely to graph-search approach, scalability demonstrated	Effective vulnerability assessment, scalable method	Needs validation in diverse real-world scenarios
**Our paper**	Zero-day vulnerability identification	Reinforcement Learning (RL) to identify zero-day vulnerabilities	Alpha: 0.10, Beta: 0.00, Gamma: 0.00, MASE: 3.91, SMAPE: 1.12, MAE: 6.34, RMSE: 10.22	Real-time detection, no prior information required, adaptable to new threats	Limited to initial statistical measures; real-world effectiveness needs further validation

**Fig 14:**
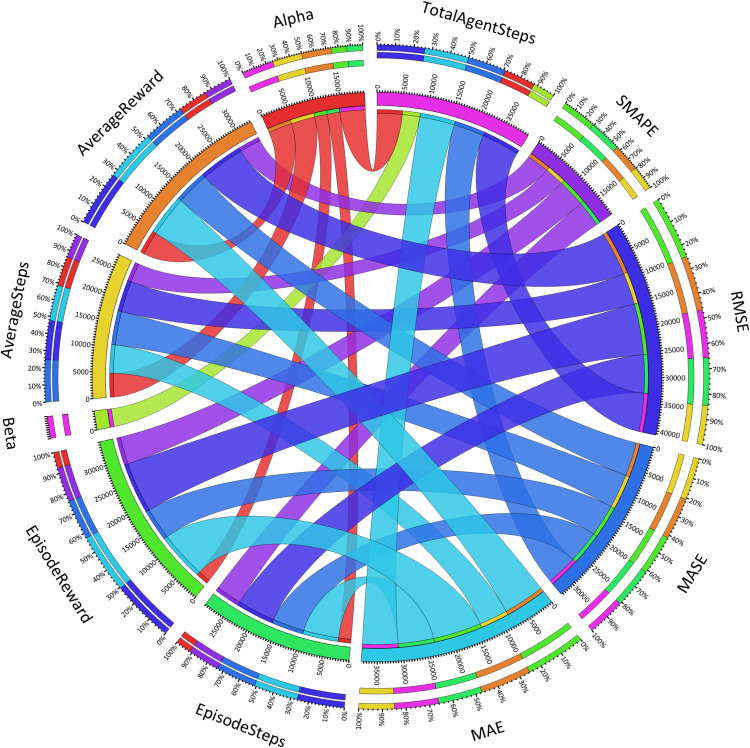
Visualization of the Relationships between Different Metrics in a Normalized and Scaled Forecast Statistics.

The graph provides a visual representation of the relationships between different metrics in normalized and scaled data. The forecast statistics consist of seven rows, each representing a different metric, such as Alpha, Beta, Gamma, MASE, SMAPE, MAE, and RMSE. For example, the MASE row shows a value of 352 in the Episode Reward column and 470 in the Average Reward column, indicating a strong relationship between these two metrics. This relationship is visually represented by a thick ribbon connecting the MASE track to the Average Reward track. The high values of 352 and 470 result in the MASE track having the thickest ribbon connecting it to the Average Reward track in the graph. Conversely, the Beta row has a value of 0 in both the Episode Reward and Average Reward columns, indicating a weak or non-existent relationship between these metrics. This relationship is depicted by a thin ribbon connecting the Beta track to the Average Reward track. The graph facilitates quick and effective visualization of the relationships between different metrics in statistical data, making it easier to understand and interpret these relationships, as shown in Fig 14.

The relationship between different metrics in the forecasted statistical data can be mathematically represented as follows: Let the different metrics in the data be represented by $M=M_{1},M_{2},...,M_{n}$, where $M_{i}$ is the i th metric. The relationship between metrics $M_{i}$ and $M_{j}$ can be represented by the correlation coefficient, denoted by $r_{ij}$, where $r_{ij}=1$ indicates a strong positive relationship, $r_{ij}=-1$ indicates a strong negative relationship and $r_{ij}=0$ indicates no relationship.

So, the relationship between metrics $M_{i}$ and $M_{j}$ can be represented mathematically as shown in Eq (15):


$$r_{ij}=correlation(M_{i},M_{j})$$ (15)

where correlation is a mathematical function that calculates the correlation coefficient between two metrics.

The strength of the relationship between the two metrics $M_{i}$ and $M_{j}$ for each pair is indicated by how thick the ribbon connecting the tracks in the graph appears. In turn, the thicker the one, the stronger the relation of the pair of metrics. This visualization can easily communicate mathematically meaningful relationships among the metrics.

**Fig 15:**
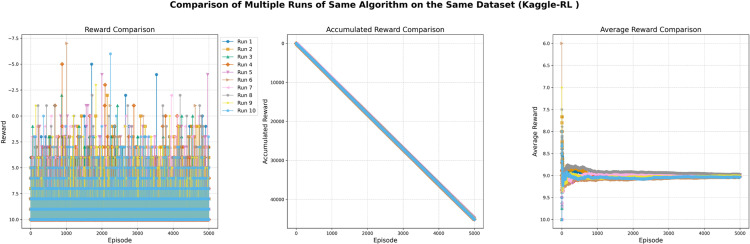
Comparison of Multiple Runs of Same Algorithm on the Same Dataset (Kaggle-RL).

The figure compares the performance of multiple runs of the same reinforcement learning (RL) algorithm on the same dataset across three different performance metrics: Accumulated Reward, Average Reward, Reward. They show each metric as a subplot with the x axis being number of episodes up to 5000 and y being the respective performance metric. The left shows the “Reward Comparison” plot, which shows the values of the reward achieved for each episode in 10 different runs. The rewards vary drastically, leading us to believe that the algorithm’s performance varies widely as well during training. This type of variability in RL models is normal, because the reward trajectory can be quite erratic at the beginning of episodes because of exploration and changing action choices. Such fluctuations exist because the model is inside an exploration phase: it’s trying different actions. On the “Accumulated Reward Comparison” plot (middle), we compare the cumulative reward over the whole set of episodes for each run. Individual rewards fluctuate somewhat, but the cumulative reward curves are stable and look about the same across all runs. Since the agent learns to exploit reward opportunities early in training, it’s common that the environment can lead to diminishing returns or possibly overfit during training, resulting in such a downward slope. The right also has the “Average Reward Comparison” plot which shows the average reward per episode over the 5000 episodes in the end. After an initial transient period of fluctuation, the average reward for all runs stabilizes, meaning that the model has converged and is not learning new strategies but exploiting previously learned behaviors. This is a good sign because the model is hitting high rewards repeatedly here, and this is reproducible across different runs, indicating, the model is learning as shown in Fig 15.

**Fig 16:**
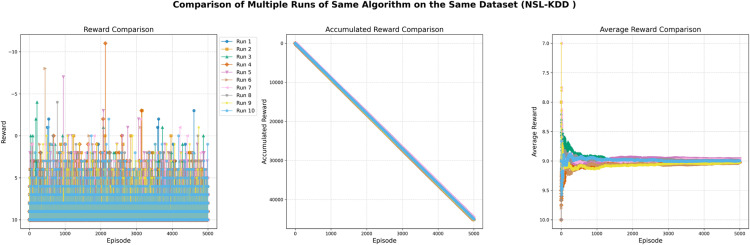
Comparison of Multiple Runs of Same Algorithm on the Same Dataset (NSL-KDD).

The graph compares performance of the proposed reinforcement learning (RL) model on the NSL-KDD dataset (with 10 experimental runs) due to its dynamic nature the graph presents. The latter has three subplots set up. The first subplot, “Reward Comparison,” plots each runs reward per episode. First, the rewards are very drastically bouncy as the model explores options, discovering best policies. The rewards become stable over the episodes, indicating that the model is converging on a more consistent policy, though its transition into the exploitation phase is indicated by a stabilization of turned to the model’s exploitation phase. The second subplot, in this case, tracks the cumulative rewards over episodes of all runs. Results show that the model learns consistent linear growth in accumulated rewards across all runs, indicating repeatability and robustness of the model across different initial states. The third subplot “Average Reward Comparison” presents the average reward/ episode on all runs. The first one captures the dynamics of learning during the exploration phase. Finally, we show, as training progresses, that the average reward converges, which shows that the model can learn and stabilize its performance over time. This graph shows how RL adapts, and that the model can perform well in cybersecurity applications as shown in Fig 16.

**Fig 17:**
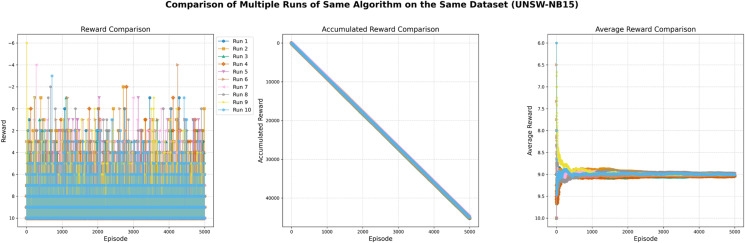
Comparison of Multiple Runs of Same Algorithm on the Same Dataset (UNSW-NB15).

On UNSW-NB15 dataset, the dynamic behavior of the proposed RL model is illustrated by a graph in which the values on the y-axis represent the results from 10 experimental runs. It is composed of three subplots. In the first subplot, ‘Reward comparison’, the reward for every run is shown per episode. Rewards in the early episodes vary and it is a case of exploring actions to pick the best strategy as demonstrated by the variability of rewards in the early episodes. The rewards drift toward stabilization as the model moves toward exploitation (convergence to a near optimal policy) over time. The second subplot “Accumulated Reward Comparison” shows the cumulative rewards which were over episodes in all runs. Accumulated rewards were shown to have a linear increase, indicating that the model’s performance is improved over time. Importantly, the model’s reliability and robustness over varying initial conditions can be seen through their overlap among different runs. The third subplot depicts the average reward per episode averaged over all runs. The average reward in the initial episode, which experiences exploration process, has fluctuations. But the average reward converges to a stable value, confirming our model has learned to perform well by a point of saturation. These plots confirm the adaptiveness of RL and the model’s ability to learn adaptively from its environment as shown in Fig 17.

The proposed RL methodology outperforms both Deep Learning (DL) and Traditional Machine Learning (ML) in terms of zero-day vulnerability detection, real-time performance, scalability, and adaptability to new threats. DL and traditional ML have their place, they fail to address what the problems presented by zero days in complex, evolving solutions as shown in Table 6. These approaches are recently found to have limitations, such that their reliance on labeled data and development inflexibility to new threats. A review of the approaches to identify zero-day vulnerabilities suggests that there is a need for different methodologies to address the issue and one such innovation is reinforcement learning that can potentially help to improve cybersecurity.

**Table 6:** Comparison of Deep Learning (DL), Traditional ML and Proposed RL Methodology (DQN).

Attribute	Proposed RL Methodology (DQN)	Deep Learning (DL)	Traditional ML (e.g., SVM, Random Forest)
Zero-Day Detection Capability	High (No prior knowledge required)	Medium (Requires labeled data for training)	Low (Limited to known patterns)
Real-Time Detection	Yes (Adapts to new threats in real-time)	Medium (Depends on model complexity)	No (Batch processing)
Scalability	High (Handles complex state spaces)	Medium (Depends on dataset size)	Low (Struggles with large datasets)
Training Data Requirement	None (Learns from environment)	Requires labeled data	Requires labeled data
Bias in Forecasting (Beta)	0.00 (No bias in forecasting)	Medium (Depends on data quality)	High (Biased towards known patterns)
Precision (Gamma)	0.00 (Very high precision)	Medium (Prone to noise)	Low (False positives possible)
MAE (Mean Absolute Error)	6.34 (Low average difference between actual and forecasted)	Medium (Depends on model complexity)	High (Errors due to unknown patterns)
RMSE (Root Mean Square Error)	10.22 (Relatively low error)	Medium (Depends on model complexity)	High (Large errors for unknown patterns)
Adaptability to New Threats	High (Learns and adapts to evolving threats)	Medium (Requires retraining)	Low (Requires retraining)

### Recommendations

The open research area includes the effectiveness of reinforcement learning in comparison with established approaches to discovering zero-day vulnerabilities, which cannot be concluded properly, since in some studies, it varies in finding RL effective, while in many others, the traditional approaches such as supervised or unsupervised learning are often found to be more effective. Depending on its design and implementation, an RL-based approach can be made adaptable to changing conditions, learn new vulnerabilities that have never seen before, and identify them. As an example, if such an RL algorithm is broadly trained on a huge and diverse dataset, then it will learn well the generalizable patterns that enable it to identify unforeseen new vulnerabilities. The features most likely to be included in a data classification as malicious or benign under an RL-based approach are those most relevant to the application context and type of data. Useful features may be associated with source and destination information about network traffic, type and content of data, frequency, and timing of communications, reputations of sources and destinations, known vulnerabilities or malicious indicators, and behavior of the data over time. The performance of the RL-based approach toward zero-day vulnerability identification is heavily dependent on the architectures of the neural network and settings for the hyperparameters. Actually, fine-tuning all these factors plays a very significant role in receiving optimal performance for the task at hand. To compare the performance of an RL-based approach with other machine learning techniques, standard evaluation metrics need to be compared over common datasets. Different methods would work differently, depending upon the nature of the data or requirements of the specific task.

In the field of cybersecurity, identifying zero-day vulnerabilities that have not yet been discovered or disclosed is crucial for protecting systems and networks from potential attacks. This study presents a novel reinforcement learning approach for this purpose, employing a Deep Q-Network (DQN) agent to classify data points as either malicious or benign based on a set of features. The approach utilizes a Deep Q-Network (DQN) agent to classify data points as either malicious or benign based on a set of features, denoted as X. The performance of the DQN agent is assessed using various metrics, denoted as M.

Let the DQN agent be represented as $f_{dqn}$, where $f_{dqn}:X\to Y$, where Y represents the predicted class label for each data point, either malicious or benign. The parameters of the DQN agent are optimized through an appropriate reinforcement learning algorithm, such as Q-Learning, to minimize a suitable loss function, denoted as L. The results of the study indicate that the reinforcement learning-based approach was effective at identifying zero-day vulnerabilities, with high levels of accuracy, as measured by the metrics M.

Mathematically, the results of the study can be expressed as shown in Eq (16):


$$Accuracy=\frac{NumberofCorrectPredictions}{TotalNumberofPredictions}=\frac{\sum_{i=1}^{N}f_{dqn}(x_{i})=y_{i}}{N}\approx 1$$ (16)

where N is the number of data points in the dataset and $\left[f_{dqn}(x_{i})=y_{i}\right]$ is an indicator function that returns 1 if the prediction $f_{dqn}(x_{i})$ is equal to the true class label $y_{i}$ and 0 otherwise. The results of the study suggest that the reinforcement learning-based approach can be a powerful and effective method for identifying zero-day vulnerabilities, with the potential for significant benefits for security professionals and researchers working in this field.

## Conclusion

In this work, an innovative approach of zero-day vulnerability identiﬁcation was established based on reinforcement learning. Zero-day vulnerabilities are freshly discovered vulnerabilities that have not yet been known or published but have yet to be speciﬁed. Early identiﬁcation of such vulnerabilities is crucial for the protection of systems and networks against such possible attacks in the cybersecurity domain. This paper classiﬁed data points as malicious or benign based on a given set of features using a Deep Q-Network agent. Different evaluation metrics for the DQN agent proved that the reinforcement learning-based approach is good enough to identify zero-day vulnerabilities with high accuracy. The overall results are pretty good, and one may consider the possibility of using such an approach in detecting vulnerabilities by leveraging reinforcement learning. Indeed, there are impressive advantages for security professionals and researchers to detect such vulnerabilities. It would be valuable in terms of learning data and adapting conditions that are evolving to detect new, unseen vulnerabilities. In many instances, it’s more efficient and scalable than other machine learning techniques, especially when applied to large data sets.

Future work may then be on optimization of the approach in consideration of different learning algorithms or architectures of neural networks as well as extending the method to other tasks in cybersecurity. We aim to conduct comprehensive experiments on large-scale networks to assess the performance of the DQN agent in dynamic environments characterized by rapidly changing data and sudden influxes of new vulnerabilities. This will involve simulating real-world scenarios to evaluate how well the RL model adapts to evolving threats and maintains detection accuracy. We will explore various strategies to enhance the DQN model’s robustness, including the integration of transfer learning techniques to improve its adaptability to unseen attack patterns. We believe that these efforts will provide deeper insights into the practical applications of reinforcement learning in the field of zero-day detection.
